# Histological diagnosis of immune checkpoint inhibitor induced acute renal injury in patients with metastatic melanoma: a retrospective case series report

**DOI:** 10.1186/s12882-020-02044-9

**Published:** 2020-09-07

**Authors:** Sebastian Hultin, Kazi Nahar, Alexander M. Menzies, Georgina V. Long, Suran L. Fernando, Victoria Atkinson, Jonathan Cebon, Muh Geot Wong

**Affiliations:** 1 Department of Renal Medicine Royal North Shore Hospital, Sydney, Australia; 2 Sydney Medical School, The University of Sydney, Sydney, Australia; 3 Westmead Institute of Medical Research, 176 Hawkesbury Road, Westmead, NSW2145 Australia; 4 Melanoma Institute Australia, The University of Sydney, Sydney, Australia; 5 Department of Medical Oncology, Royal North Shore Hospital and Mater Hospital, Sydney, Australia; 6 Department of Immunopathology, NSW Health Pathology North, Sydney, Australia; 7 Princess Alexandra Hospital, Brisbane, Australia; 8 University of Queensland, Brisbane, Australia; 9 Olivia Newton-John Cancer Research Institute at Austin Health, Heidelberg, Australia; 10 The George Institute for Global Health, Sydney, Australia

**Keywords:** AKI, Glomerulonephritis, Immunology, Renal biopsy, Tubulo interstitial nephritis, Immune checkpoint inhibitor

## Abstract

**Background:**

Immune checkpoint inhibitors (ICI) have become the standard of care in many oncological conditions but are associated with a spectrum of renal immune-related adverse events (IrAEs). We aimed to describe the spectrum, histology, management and outcomes of renal IrAE in patients with metastatic melanoma undergoing ICI therapy.

**Methods:**

We conducted a retrospective review of 23 patients with a diagnosis of metastatic melanoma treated with ICI between January 2017 and April 2019 who developed a renal IrAE. Baseline demographic data, biochemical and histopathological results, management and outcomes were analyzed.

**Results:**

The majority of patients who developed renal irAE were male and received combination immunotherapy. The median time of onset from initiation of ICI therapy to renal IrAE was 4 months. 52% of the treated renal IrAE had histopathologically confirmed renal IrAE. The most common histological pattern of injury was acute tubulo-interstitial nephritis (92%). One patient developed anti-GBM disease with non-dialysis dependent stage 5 CKD. In tubulointerstitial injury, there was no association between peak creatinine, renal recovery and histologically reported inflammation or fibrosis. Patients with renal IrAE demonstrated persisting renal dysfunction at 3, 6 and 12 months with a mean baseline, 3 and 12 month creatinine of 90.0 μmol/L, 127.0 μmol/L and 107.5 μmol/L respectively.

**Conclusion:**

Renal IrAE is most commonly attributable to steroid responsive acute tubulointerstitial nephritis. The outcome of rarer pathologies such as anti-GBM disease may be adversely affected by a delayed diagnosis. There is persisting renal dysfunction following an episode of renal IrAE that may have impact on future renal and overall survival outcomes.

## Background

Immunotherapy is now the standard of care for many patients with cancer, offering the chance of sustained cancer control and possible cure [1]. Ipilimumab, an anti-cytotoxic T-lymphocyte-associated protein 4 (CTLA-4) monoclonal antibody was the first drug to improve survival for metastatic melanoma [2]. Subsequently, anti-Programmed cell Death protein (PD-1) and PD-1 Ligand (PD-L1) antibodies including nivolimumab and pembrolizumab demonstrated impressive activity in several cancer types including metastatic melanoma, non-small cell lung cancer, urothelial cancer, head and neck cancer, Hodgkin lymphoma, and microsatellite instability-high or mismatch repair deficient solid tumors such as metastatic colorectal carcinoma [2–11]. Combined immune checkpoint inhibition with nivolumab and ipilimumab has demonstrated improved activity in metastatic melanoma and advanced renal cancer compared to nivolumab alone or targeted therapy [10, 12].

Immune checkpoint inhibitors (ICIs) are associated with a spectrum of inflammatory side effects termed immune-related adverse events (IrAEs) [13]. These IrAEs are common, can affect almost any organ with varying severity, and occur with a reported incidence of severe adverse events of 0.5–13% [13, 14]. IrAE most commonly affecting the skin, gastro-intestinal, endocrine and respiratory systems, but can involve any organ. Immunotherapy related acute kidney injury (AKI) or renal IrAE was reported in early clinical trials with an overall AKI incidence rate of 2.1% from PD-1 ICI therapy alone and 5% with combination therapy [15–17]. Case reports suggest that the most common renal IrAE is acute interstitial nephritis (AIN), with or without granulomas, but focal segmental glomerulosclerosis (FSGS), thrombotic microangiopathy (TMA), immune-complex glomerulonephritis, drug induced lupus, anti-glomerular basement membrane (anti-GBM) glomerulonephritis, and minimal change disease (MCD) have also been reported [17–22]. The clinical presentation, management and outcomes of renal IrAE is highly variable, without consensus for the role of renal biopsy. We present a retrospective review of renal IrAE from immunotherapy in metastatic melanoma patients from major cancer institutes in Australia.

## Methods

Patients undergoing immunotherapy for metastatic melanoma at our oncology service network and national collaborators from January 2017 to April 2019 were eligible for inclusion. Patients with renal IrAE (defined as individuals with AKI attributed to immunotherapy with a greater than 50% elevation from baseline creatinine and/or a rise in creatinine by greater than 26 μmol/L, corresponding to a Kidney Disease Improving Global Outcomes (KDIGO) stage 1 or higher AKI) and referred to nephrology were included. All subjects provided informed consent and the study was conducted with institutional ethical review board approval.

Baseline demographics, biochemistry, and treatment details were collected from electronic database and clinical records. Pre-existing Chronic Kidney Disease (CKD) was defined as an eGFR < 60 mL/min/1.73m^2^. Renal function recovery was defined as recovery of renal dysfunction or return of creatinine to pre-AKI baseline. Investigations for renal IrAE consisted of urinalysis (microscopy and proteinuria/albuminuria quantification), renal ultrasonography, and autoimmune screen (Anti neuronal antibodies (ANA), Extractable Nuclear Antigen antibodies (ENA), anti double stranded DNA antibodies (dsDNA), anti-neutropil cytoplasmic antibodies (ANCA), including proteinase 3 (PR3) and Myeloperoxidase (MPO) quantification, anti glomerular membrane antibodies (anti-GBM), complement C3,C4 level). Monoclonal gammopathy screen was performed if indicated clinically. Patients were subsequently considered for renal biopsy based on i) severity of AKI, ii) rapidity of improvement on oral prednisolone therapy, iii) underlying co-existing factors including prior history of CKD, antiplatelet/anticoagulant therapy and comorbidities.

Renal outcomes assessed were: [1] AKI by KDIGO criteria [2]; renal function (creatinine and eGFR based on CKD-Epi) at 3, 6 and 12 months from the time of diagnosis of AKI [3]; new or worsening CKD defined as renal dysfunction compared to baseline at 3 months post IrAE related AKI. Oncological outcomes reported were [1] discontinuation of immunotherapy [2]; IrAE affecting other organs [3]; Best response to treatment as per RECIST version 1.1 (Response Evaluation Criteria In Solid Tumor) defined as complete response (CR), partial response (PR), stable disease (SD) or progressive disease (PD).

### Statistical analysis

Normally distributed variables are expressed as mean and standard deviation. Background biopsy fibrosis was grouped into absent/mild compared to moderate/severe and severity of interstitial inflammation was grouped into mild/moderate compared to severe based on histopathological reporting. Paired t-tests were used for group comparisons. *P* > 0.05 was considered statistically significant.

## Results

### Population and baseline characteristics

A total of 23 patients with metastatic melanoma were included and baseline demographic details are summarized in Table 1. The mean age was 67 years (SD 13.6. range 36–88 years. IQR 22) and majority were male (91%; 21/23). 22% (5/23) of the cohort had no cardiovascular or metabolic comorbidities (Table 1). In the remaining patients, hypertension was reported in 66% (12/18), diabetes mellitus in 50% (9/18), and vascular disease (ischaemic heart disease, peripheral vascular disease or previous stroke) in 28% (5/18). 22% (5/23) of patients received proton pump inhibitors at the time of renal IrAE. 9% (2/23) patients had received antibiotics in the month leading to renal IrAE. Although confounders, initiation of these drugs were temporally unrelated to the episode of AKI and less likely to be causal.

**Table 1:** Patient demographic data, past history and individual renal and oncological outcomes

Kidney Biopsy	Age range (yr)	Gender	BMI (kg/m^**2**^)	Comorbidities	Medications prior to renal IrAE	Immunotherapy regimen	Renal function 3m	Oncological outcome
Yes	40-49	Male	25.1	Nil	Nil	Ipilimumab + Nivolimumab	CKD 2	CR
Yes	50-59	Male	25.6	HTN	Metoprolol 50mg twice daily, amdodipine 10mg daily	Ipilimumab + Nivolimumab	CKD 3a	CR
Yes	60-69	Male	26.2	Nil	Nil	Ipilimumab + Nivolimumab	CKD 2	PR
Yes	70-79	Male	29.2	HTN, BPH, nephrolithiasis, acquired hypopituitarism	Thyroxine 100mcg daily, Prednisolone 10mg daily.	Ipilimumab + Nivolimumab	CKD 3a	PR
Yes	70-79	Male	27.5	HTN, dyslipidaemia, IHD, BPH, GORD	Quinapril 5mg daily, rosuvastatin 20mg daily, allopurinol 200mg daily, rabeprazole 20mg daily.	Pembrolizumab + Epacadostat	CKD 3a	PD
Yes	70-79	Male	24.6	HTN, IHD, DM, Diverticular disease.	Amitriptyline 50mg daily, telmisartan 40mg daily.	Ipilimumab + Nivolimumab	CKD 3a	PD
Yes	70-79	Male	29.2	IHD, BPH, HTN	Aspirin 100mg daily, clopidogrel 75mg daily, atorvastatin 20mg daily.	Ipilimumab + Nivolimumab	CKD 3b	PD
Yes	60-69	Female	41.2	HTN, GORD, rectal cancer.	Perindopril 2.5mg daily, rabeprazole 20mg daily.	Ipilimumab + Nivolimumab	CKD 3a	CR
Yes	70-79	Male	28.5	Acquired hypopituitarism	Hydrocortisone twice daily, thyroxine 100mg daily	Ipilimumab + Nivolimumab	CKD 4	CR
Yes	50-59	Male	26	Previous pulmonary embolus	Nil	Ipilimumab + Nivolimumab	CKD 5	CR
Yes	80-89	Male	23	Idiopathic cardiomyopathy. PMR.	Data not available	Pendrolizumab	CKD 3b	SD
Yes	30-39	Male	27	Nil	Nil	Ipilimumab + Nivolimumab	CKD 2	PD
No	40-49	Male	35.9	HTN, Obesity, Depression	Perindopril 5mg daily, venlafaxine 75mg daily	Ipilimumab + Nivolimumab	CKD 2	CR
No	70-79	Male	28.1	T2DM, HTN, IHD, dyslipidaemia, smoker, BPH	Quinapril 5mg daily, rosuvastatin 20mg daily, metformin 1000mg daily, gliclazide 60mg daily, dapaglifozin (unknown dose) daily.	Ipilimumab + Nivolimumab	CKD 3a	PD
No	70-79	Female	31.6	HTN, dyslipidaemia	Data not available	Ipilimumab + Nivolimumab	CKD 3a	PD
No	70-79	Male	33	T2DM, HTN, dyslipidaemia, IHD, PVD, BPH, haemochromatosis, EtOH excess	Aspirin 100mg daily, clopidogrel 75mg daily, perindopril 5mg daily, hydrochlorothiazide 12.5mg daily, amlodipine 10mg, atorvastatin 40mg daily.	Ipilimumab + Nivolimumab	CKD 3b	CR
No	50-59	Male	31.3	Nil	Data not available	Pendrolizumab	Full recovery	PR
No	60-69	Male	27.8	HTN, CVA, cardiomyopathy.	Bisoprolol 5mg daily	Ipilimumab + Nivolimumab	CKD 3a	PR
No	70-79	Male	24.9	HTN, Seizures,	Aspirin 100mg daily, irbesartan 150mg daily, alendronate, simvastatin 40mg daily, Omeprazole 20mg daily, monoxidine 200mcg daily.	Ipilimumab + Nivolimumab	CKD 3b	CR
No	60-69	Male	N/A	Nil	Nil	Ipilimumab + Nivolimumab	CKD 2	PR
No	70-79	Male	N/A	IHD, T2DM, dysllipidaemia.	Candesartan 32mg daily, aspirin 100mg daily, clopidogrel 75mg daily, simvastatin 40mg daily, propanolol 80mg daily, Ranitidine 150mg daily	Ipilimumab + Nivolimumab	CKD3b	CR
No	70-79	Male	27.5	Bladder Ca (sx), IHD, AF, DVT	Aspirin 100mg daily, nicorandil 10mg daily, rosuvastatin 20mg.	Pembrolizumab	CKD 3a	CR
No	80-89	Male	N/A	Bullous pemphigoid, DM, DVT, PVD, AF	Apixaban 2.5mg twice daily, metformin 1000mg daily, rosuvastatin 20mg daily, pantoprazole 40mg daily, gliclazide 60mg daily.	Nivolimumab	CKD 3b	PD

HTN – hypertension, IHD – Ischemic Heart Disease, PVD – peripheral vascular disease, BPH – Benign prostatic hypertrophy, GORD – Gastro Oesophageal Reflux Disease, PE – pulmonary embolus, CA – cancer, T2DM – Type 2 diabetes mellitus, EtOH – alcohol excess, CVA – stroke, AF – atrial fibrillation, DVT – deep venous thrombosis, CKD – chronic kidney disease, CR – Complete remission. PR – partial remission. SD – Stable disease. PD = progressive disease

The mean baseline creatinine was 90 μmol/L (Range 53–125 μmol/L. SD 18.5) and eGFR 73 mL/min/1.73m^2^ (range 48–90 SD 15.0). Four patients had pre-existing CKD (eGFR < 60 mL/min/1.73m^2^) at the time of ICI commencement – all were classed as CKD stage 3a. The most common immunotherapy regimen was ipilimumab and nivolimumab (74%; 17/23). One patient received the combination of ipilimumab and pembrolizumab; four patients received single agent pembrolizumab; one patient received PD-1 monotherapy. Twelve patients underwent diagnostic renal biopsy; the remaining patients (*n* = 11) satisfied clinical and biochemical criteria for renal IrAE.

### Biochemistry, urinalysis, and autoimmune profile

Results of investigations at baseline and at onset of renal IrAE are summarized in Table 2. Less than half of the patients (43.5%; 10/23) had baseline urinalysis available prior to the diagnosis of renal IrAE. At AKI, 30% (7/23) patients had pyuria without bacterial growth on culture with a previously bland urine sediment. A further two patients had pyuria with a positive urine culture. 17% (4/23) of patients had microscopic haematuria and 1 patient macroscopic hematuria in the context of previous bladder cancer and was investigated for recurrence. 30% (7/23) of patients had proteinuria (urine protein:creatinine ratio > 30 mg/mmol or urine albumin:creatinine ration > 3.5 g/mol) at the time of diagnosis of AKI. Four patients had positive ANA titres (3 patients with low titre < 1:80 with one patient with a titre 1:2560. All patients had negative ENA and dsDNA antibodies). One patient had a positive pANCA but absent anti- MPO and PR3 antibodies. None of these patients had clinical symptoms suggestive of systemic autoimmune disease or vasculitis. One patient with markedly elevated antiGBM antibody titres (614 U/mL. normal < 5 U/mL) and was confirmed on kidney biopsy as anti-GBM glomerulonephritis and treated accordingly.

**Table 2:** Urinalysis and autoimmune screen results

Patient	Baseline Urinalysis				Urinalysis at AKI				Autoimmune screen
	Haematuria	Pyuria	Proteinuria	Culture / Casts	Haematuria	Pyuria	Proteinuria	Culture / Casts	
1	-	-	-	-	-	-	-	-	negative
2	NA	NA	NA	NA	-	-	-	-	negative
3	-	+	-	-	-	-	-	-	negative
4	NA	NA	NA	NA	-	+	-	-	negative
5	-	+	-	-	-	+	+	-	Not available
6	NA	NA	NA	NA	-	+	+	-	Not available
7	-	-	-	-	-	+	+	Hyaline casts	ANA 1:2560.
8	NA	NA	NA	NA	+	+	-	E. *cloacae* UTI	Not available
9	-	-	-	-	-	-	NA	-	negative
10	NA	NA	NA	NA	+	+	N/A	E. Coli infection	anti GBM 614u/mL
11	-	-	-	-	-	-	+	-	ANA 1:80
12	NA	NA	NA	NA	+	+	+	-	negative
13	-	-	-	-	-	-	+	-	negative
14	+	-	-	-	-	+	-	-	negative
15	-	-	-	NA	+	+	+	-	negative
16	+	Nil	+	-	+	+	+	Hyaline casts	ANA 1:40
17	-	-	-	NA	+	+	-	-	Not available
18	-	-	-	-	-	-	-	-	negative
19	-	-	-	-	-	-	-	-	negative
20	+	+	NA	-	+	+	+	-	Negative
21	NA	NA	NA	NA	+	+	-	-	pANCA+ve MPO/PR3 negative
22	+	+	-	-	-	+	-	-	negative
23	-	-	+	NA	-	-	+	-	ANA 1:80

NA – not available. Haematuria – > 10 red cells per high power field on microscopy or > 1+ on urine dipstick. Pyuria – > 10 white cells per high power field on microscopy or > 1+ on urine dip stick. Proteinuria – raised albumin/protein:creatinine ratio or > 1+ protein on urine dip stick. ANA – Anti Nuclear Antibody; ANCA – Anti Neutrophil Cytoplasmic Antibody; antiGBM – Glomerular Basement Membrane; MPO – Myeloperoxidase; PR3 – Proteinase 3

### Renal IrAE management and outcome

The median time from immunotherapy initiation to renal IrAE was 4 months (range 2–24 months). The majority of patients had IrAE affecting multiple organs during the course of ICI therapy (74%, 17/23). The most common IrAE excluding nephritis was colitis (7/23, 30% any grade, 13% grade 3), hepatitis (6/23, 26% any grade, 13% grade 3), endocrinopathies (4/23, 17% with grade 2 thyroiditis accounting for 50% of cases and grade 2 hypophysitis for the other 50%) and pneumonitis (2/23, 9% any grade. 5% grade 3).

There was no significant difference in baseline creatinine (prior to immunotherapy initiation) between patients who underwent biopsy and those who did not (84.8 μmol/L [SD 19.4]) vs 92.6 μmol/L [SD 22.6], *p* = 0.18). The biopsy group had a higher mean peak creatinine compared to the non-biopsy group (429.9 μmol/L [SD 341.7] vs 234.5 μmol/L [SD 166.4] respectively) reflecting more severe kidney injury in patients referred for biopsy. Patients who underwent biopsy, had the procedure performed within 2–7 days of AKI, depending on proceduralist availability and patient optimization prior to procedure, except for one patient where biopsy was undertaken 2 weeks post AKI due to persistent renal dysfunction despite immunosuppressive therapy.

ICI therapy was discontinued in 78% of patients (18/23 patients). All patients were commenced on oral prednisolone 1–1.5 mg/kg at diagnosis of renal IrAE. Eight patients (three in the non-biopsy group and five in the biopsy group) in addition received pulse methylprednisolone 500-1000 mg daily for three consecutive days with step down to oral prednisolone 1–1.5 mg/kg based on the acuity and severity of renal dysfunction. Two patients in the biopsy group required additional immunosuppression – one patient with biopsy proven tubulointerstitial nephritis received mycophenolate maintenance in the context of steroid non responsiveness; a second patient was treated for ICI related anti-GBM disease as described below.

The majority of patients showed evidence of improvement of renal function following corticosteroid therapy, with kidney function stabilization by three months (127.0 μmol/L [SD 35.1] *n* = 20), with only marginal subsequent improvement at 6 and 12 months (Table 3). On comparison of the degree of the difference between patients’ baseline creatinine and creatinine at 3, 6 and 12 months there was evidence of persisting kidney impairment in the majority of patients. At 3 months there was a statistically significant difference compared to baseline creatinine (mean 89.8 μmol/L vs 127.0 μmol/L. *p*-value < 0.001). The difference persisted at 6 months (mean 89.8 μmol/L vs 132.0 μmol/L. *p*-value 0.002) and at 12 months (mean 89.8 μmol/L vs 107.5 μmol/L. p-value 0.009). Similarly, comparison of baseline eGFR (73 mL/min/173m^2^) and eGFR at 3 months (54 mL/min/173m^2^. *p*-value < 0.001) and 6 months (eGFR 53 mL/min/173m^2^. p-value 0.002) indicated persistent renal dysfunction, which was statistically significant. This difference was lost at 12 months (63 mL/min/173m^2^. p-value 0.068). At 3 months only one patient had returned to their baseline kidney function, with five patients classed as KDIGO CKD stage II, nine patients had CKD stage IIIa, six patients had CKD stage IIIb and one patient CKD stage IV (Table 1).

**Table 3:** Renal function at baseline, 3, 6 and 12 months following renal IrAE

	n	Renal function	SD	***p***-value in relation to baseline
**Baseline Creatinine (μmol/L)**	23	89.8	18.5	N/A
**Baseline eGFR (mL/min/1.73m**^**2**^**)**		73	15	N/A
**Peak Creatinine (μmol/L)**	23	336.5	285	*p* = <0.001
**Peak eGFR (mL/min/1.73m**^**2**^**)**		24	12.4	*p* = <0.001
**Creatinine at 3m (μmol/L)**	21	127.4	35.1	*p* = <0.001
**eGFR at 3m (mL/min/1.73m**^**2**^**)**		54	17.9	*p* = <0.001
**Creatinine at 6m (μmol/L)**	16	132	63.8	*p* = 0.002
**eGFR at 6m (mL/min/1.73m**^**2**^**)**		54	22.7	*p* = 0.002
**Creatinine at 12m (μmol/L)**	11	107.5	20.9	*p* = 0.009
**eGFR at 12m (mL/min/1.73m**^**2**^**)**		63	18.1	*p* = 0.068

eGFR – estimated glomerular filtration rate. N/A – not available. m – month

### Renal histology

More than half of the cohort (52%, 12/23) underwent kidney biopsy. Results are summarized in Table 4. The most common reasons for not conducting a biopsy was frailty, high comorbidity burden or rapid improving renal function with steroid therapy. There were no complications related to the kidney biopsy.

**Table 4:** Renal biopsy histopathology results

Patient	Gloms	Glomeruli	Tubules	Interstitium	Vascular	BG atrophy/fibrosis	IF	EM
**1**	16	Normal	Tubulitis	Marked inflammation	Mild to moderate afteriosclerosis	Mild	Negative	Dense interstitial lymphocytic and neutrophilic infiltrate and tubulitis.
**2**	35	Normal	patchy florid tubulitis	Patchy inflammation. mixed multinucleate giant cell granulomas	granulomatous inflammation along vessels	None	Negative	Patchy moderate interstitial fibrosis and oedema. lymphocytes infiltrate and few plasma cells. focal tubulitis.
**3**	18	2/18 sclerosed. Mild chronic ischaemic change	patchy moderate tubulitis	Mild inflammation	moderate patchy arteriosclerosis and intimal thickening.	Severe	Negative	Interstitial fibrosis and atrophy with ischaemic glomerular change. Tubular intramural lymphocytes.
**4**	14	2/14 sclerosed. Normal	Occasional tubulitis.	Patchy dense foci of moderate inflammation	moderate arteriosclerosis.	Moderate	IgM - 1+ granular mesangial stain.	Moderate interstitial fibrosis and tubular atrophy. Few interstitial lymphocytes
**5**	18	2/18 sclerosed. chronic ischaemic change	No tubulitis	Patchy inflammation.	focal arteriolesclerosis.	Moderate	C3 - trace granular mesangial staining.	Mild patchy interstitial fibrosis and tubular atrophy. Mild focal lymphocytic infiltrate with few tubules intramural lymphocytes.
**6**	8	2/8 sclerosed. chronic ischaemic change	Tubulitis	Moderate to severe inflammation	moderate arteriosclerosis and intimal thickening	Mild	Negative	Patchy moderate interstitial fibrosis and oedema accompanied by a lymphocytic infiltrate with some neutrophils.
**7**	20	Normal	Tubulitis	Patchy moderate to severe inflammation	moderate arterisclerosis and mild arteriolar change	Mild	Lamda and kappa uptake in intraluminal material. weak C3 interstitial staining,	Predominance of acute tubulointerstitial nephritis. Sub epithelial humps and mesangial dense deposits.
**8**	11	Hypertensive change	Tubulitis	Diffuse moderate inflammation	moderate arterial fibroelastic intimal thickening.	None	Focal mesangial staining IgM.	N/A
**9**	11	Normal	Tubulitis	Diffuse severe inflammation	Normal	None	Granular deposition of IgA and C3 in mesangial region.	mesangial hypercellularity.
**10**	8	1/8 sclerosed	tubular injury	mild to moderate inflammation	mild interstitial arterial fibrosis	Mild	Negative	Patchy interstitial fibrosis and tubular atrophy. diffuse interstitial lymphocytic infiltrate.
**11**	18	2/18 sclerosed. chronic ischaemic change	Tubulitis and associated non caseiting granulomas	Non-caseating granulomas. Mod-Severe inflammation	Normal	Mild	Negative	Patchy interstitial fibrosis and tubular atrophy.
**12**	31	Necrotising cellular glomerular crescents	tubular injury and tubulitis	moderate inflammation	Normal	Mild	Linear IgG, Kappa, lamda, fibrinogen. No granular staining.	Cellular crescents. No dense deposits of fibrils

*N/A* electron microscopy report was not available in 1 patient

#### Acute tubulointerstitial nephritis

The majority of renal biopsies (92%, 11/12) displayed acute tubulointerstitial nephritis with evidence of interstitial inflammation ranging from mild to severe without glomerular abnormality. Two patients showed granulomatous interstitial inflammation, with one of these showing prominent peri-vascular inflammation. There was no correlation between the degree of reported interstitial inflammation and the severity of AKI (peak creatinine in mild/moderate interstitial inflammation 413.6 μmol/L vs 452.8 μmol/L in severe interstitial inflammation. *p*-value 0.47) or creatinine at 3 months (creatinine in mild/moderate interstitial inflammation 116.8 μmol/L vs 145.6 μmol/L in severe interstitial inflammation. p-value 0.13).

25% of patients (3/12) had moderate/severe background tubular atrophy and interstitial fibrosis with remaining patients classed as mild or absent background changes (Table 4). There was no observed correlation between reported background atrophy and fibrosis on histology and the severity of AKI (peak creatinine in absent/mild fibrosis 487.7 μmol/L vs 256.7 μmol/L in moderate/severe fibrosis. *p*-value 0.17) or creatinine at 3 months (creatinine in absent mild/fibrosis 136.6 μmol/L vs 111.7 μmol/L in moderate/severe. p-value 0.19).

#### Anti-GBM glomerulonephritis

One patient had histopathological and direct immunofluorescence changes consistent with anti-GBM disease, involving > 95% of the glomeruli. The patient presented with severe AKI with uremia, with a peak creatinine of 1382 μmol/L, hyperkalaemia (7.3 mmol/L) and uncompensated metabolic acidosis (pH 7.26, pCO2 35.6 mmHg, HCO3- 15 mmol/L) warranting urgent hemodialysis. He had received PD-1 monotherapy followed by ipilimumab and nivolimumab combination therapy for one year (AKI 51 weeks post ICI initiation) with complete oncological response. The diagnosis of anti-GBM glomerulonephritis was suspected following an elevated anti-GBM antibody level (614 U/mL) and confirmed on biopsy on day 7. The patient underwent plasma exchange and pulse steroid therapy with tapering oral prednisolone and oral cyclophosphamide. His renal function recovered after 5 months of intermittent haemodialysis. At 6 months follow-up post renal IrAE, the patient had persistent renal dysfunction (KDIGO CKD stage IV), had been weaned off all immunosuppression and had an ongoing complete oncological response.

### Oncological outcomes

A significant proportion of patients showed complete or partial response to ICI therapy - 44% (10/23 patients) and 26% (6/23 patients) respectively. 30% (7/23 patients) had progressive disease and one patient stable disease. Out of these, five patients were rechallenged with ICI therapy with the remaining 2 transitioning to combination dabrafenib and trametinib. Out of the five rechallenged patients two received single agent nivolimumab, one patient received single agent ipilimumab and two patients received pendrolizumab. There was no recurrence of renal IrAE post rechallenge.

Six patients died during the study period - four patients of progressive disease, one patient following an episode of sepsis overseas and one patient of unknown cause having been lost to follow-up.

## Discussion

Our case series, the largest reported renal IrAE in Australia, illustrates that the most common pattern of histopathological injury in renal IrAE is tubulointerstitial nephritis. There was no observed correlation between reported histopathology and the severity of renal injury or subsequent renal recovery. In addition, we have demonstrated persistent renal dysfunction in the majority of patients with renal IrAE at 12 months post renal IrAE.

Previous case series have highlighted variable time frames to onset of renal IrAE, which was confirmed in our study [17, 23]. Although, male gender was disproportionately represented in our cohort, this may represent a higher incidence of melanoma in males and selection bias due to small sample size.

Approximately 70% of patients in our study had an abnormal urinalysis, the most common abnormality being pyuria, microscopic hematuria and proteinuria. Consistent with previous reports, the clinical utility of abnormal urinary sediment in guiding diagnostic work-up, decision to proceed to biopsy or treatment remains uncertain [17]. Noting, however, the importance of screening for a glomerular pattern of injury, urinalysis remains an essential tool. In our case series, only 40% of patients had urinalysis prior to ICI initiation. Inability to confidently identify de-novo abnormality in the urinary sediment due to no baseline urine being available, can lead to inaccurate decisions surrounding biopsy.

Histopathological information associated to renal IrAE, remains insufficiently studied, with most information on renal IrAE derived from post hoc analysis of large randomized controlled oncology trials or from case series reports [17–22, 24] . This is at least partly due to the reluctance of performing a kidney biopsy, due to perceived risk and little clinical benefit. Although currently no widely accepted scoring system exists for quantification of tubulo-interstitial nephritis related to ICI therapy, kidney biopsy has the potential to provide an estimation on the severity of renal inflammation as well as chronic background changes that can provide useful information in guiding clinical decisions. Although, our study showed no correlation between the degree of reported background atrophy and fibrosis, the severity of interstitial inflammation, and the degree of renal recovery, it is important to note that small sample size and unavailability of a robust scoring system may have confounded the results. All patients in our study received steroid therapy prior to biopsy, raising the possibility of masking and under appreciating the severity of renal IrAE both biochemically and histologically. Furthermore, one patient in our case series had severe anti-GBM disease, in which a timely combination of histological and biochemical diagnosis altered the clinical course of this life-threatening disease. This highlights the importance of a robust screening process to identify patients with de-novo haematuria and proteinuria or with rapidly progressive AKI, where biopsy will provide crucial histological information to guide acute and short-medium termed management. A widely accepted scoring system may provide further guidance on the short-, medium- and long-term renal prognosis by quantifying the degree of chronic damage as well as guide decision to re-challenge the patient with ICI therapy depending on the degree of interstitial inflammation and rapidity of renal recovery.

Finally, we have demonstrated significant persisting renal insufficiency in patients following AKI attributed to immunotherapy with the majority of patients showing signs of persisting renal dysfunction at 3 months, 6 months and 12 months. This is of special interest as these patients have significantly improved mortality with an overall 3 and 5 year survival of 58 and 53% respectively, in a malignant condition that previously had a limited life expectancy [12, 25, 26].

Our study has several limitations. Our study is small and retrospective, with only cases referred to our nephrology service with concerns of renal IrAE included, limiting generalizability and possibly underestimating true incidence of ICI related AKI. Half of the diagnosed renal IrAE cases also had no kidney biopsy to confirm diagnosis. Although a larger prospective study is the most ideal scenario to corroborate our observations, it is a challenging task considering the low incidence of renal IrAEs and regional differences in nephrology practices.

## Conclusion

In summary, renal IrAEs are important toxicities with immunotherapy, likely to become more prevalent as the use of immunotherapy increases. Consistent with other reported case series, most biopsy proven renal IrAEs are steroid responsive acute interstitial nephritis, although other renal IrAEs have been reported in which early diagnosis and treatment has been shown to alter the course of the disease. Due to the selective bias of our study, however, other causes of kidney injury may have been missed. Nephrologists should have a high index of suspicion and a low threshold to obtain histological diagnosis in cases of rapidly progressive AKI with or without an active urinary sediment in patients undergoing ICI. Further histopathological study is needed, including the development and adoption of an accepted scoring system for tubulointerstitial disease that may aid in the acute management, immunosuppression wean and decision to rechallenge patients with ICI following a renal IrAE.

We have also demonstrated evidence of persistence of renal dysfunction following renal IrAE. With improved long-term survival of metastatic melanoma patients due to ICI therapy, the burden of chronic disease may well become a factor in the long-term management of metastatic melanoma patients. Further study is required to determine this observation generalizability and whether renal IrAE constitutes a significant risk factor for the development of CKD.

## Data Availability

The datasets used and/or analysed during the current study are available from the corresponding author on reasonable request.

## Abbreviations

AKI: Acute Kidney Injury
CKD: Chronic Kidney Disease
eGFR: estimated glomerular filtration rate
ICI: Immune Checkpoint Inhibitor
IrAE: Immune related Adverse Event
PD-1: Programmed Cell Death Protein 1
CTLA-4: Cytotoxic T-lymphocyte-associated protein 4
AIN: Acute interstitial nephritis
ATIN: Acute tubulo-interstitial nephritis
TMA: Trombotic microangiopathy
FSGS: Focal Segmental Glomerulosclerosis
MCD: Minimal Change Disease
anti-GBM: Anti glomerular basement membrane
ANA: Anti neuronal antibodies
ENA: Extractable Nuclear Antigen antibodies
dsDNA: double stranded DNA
ANCA: anti-neutrophil cytoplasmic antibodies
PR3: proteinase 3
MPO: Myeloperoxidase
CR: Complete oncological response
PR: partial oncological response
SD: stable oncological disease
PD: progressive oncological disease

## References

[1] Thallinger C, Fureder T, Preusser M, Heller G, Mullauer L, Holler C (2018). Review of cancer treatment with immune checkpoint inhibitors : current concepts, expectations, limitations and pitfalls. Wien Klin Wochenschr.

[2] Hodi FS, O'Day SJ, McDermott DF, Weber RW, Sosman JA, Haanen JB (2010). Improved survival with ipilimumab in patients with metastatic melanoma. N Engl J Med.

[3] Robert C, Long GV, Brady B, Dutriaux C, Maio M, Mortier L (2015). Nivolumab in previously untreated melanoma without BRAF mutation. N Engl J Med.

[4] Schachter J, Ribas A, Long GV, Arance A, Grob JJ, Mortier L, et al. Pembrolizumab versus ipilimumab for advanced melanoma: final overall survival results of a multicentre, randomised, open-label phase 3 study (KEYNOTE-006). Lancet (London, England). 2017;390(10105):1853–62. 10.1016/S0140-6736(17)31601-X 28822576

[5] Robert C, Schachter J, Long GV, Arance A, Grob JJ, Mortier L (2015). Pembrolizumab versus Ipilimumab in Advanced Melanoma. N Engl J Med.

[6] Ansell SM, Lesokhin AM, Borrello I, Halwani A, Scott EC, Gutierrez M (2015). PD-1 blockade with nivolumab in relapsed or refractory Hodgkin's lymphoma. N Engl J Med.

[7] Overman MJ, McDermott R, Leach JL, Lonardi S, Lenz HJ, Morse MA (2017). Nivolumab in patients with metastatic DNA mismatch repair-deficient or microsatellite instability-high colorectal cancer (CheckMate 142): an open-label, multicentre, phase 2 study. The Lancet Oncology.

[8] Ferris RL, Blumenschein G, Fayette J, Guigay J, Colevas AD, Licitra L (2016). Nivolumab for recurrent squamous-cell carcinoma of the head and neck. N Engl J Med.

[9] Reck M, Rodriguez-Abreu D, Robinson AG, Hui R, Csoszi T, Fulop A (2016). Pembrolizumab versus chemotherapy for PD-L1-positive non-small-cell lung Cancer. N Engl J Med.

[10] Larkin J, Hodi FS, Wolchok JD (2015). Combined Nivolumab and Ipilimumab or Monotherapy in untreated melanoma. N Engl J Med.

[11] Le DT, Uram JN, Wang H, Bartlett BR, Kemberling H, Eyring AD (2015). PD-1 blockade in tumors with mismatch-repair deficiency. N Engl J Med.

[12] Wolchok JD, Chiarion-Sileni V, Gonzalez R, Rutkowski P, Grob JJ, Cowey CL (2017). Overall survival with combined Nivolumab and Ipilimumab in advanced melanoma. N Engl J Med.

[13] Champiat S, Lambotte O, Barreau E, Belkhir R, Berdelou A, Carbonnel F (2016). Management of immune checkpoint blockade dysimmune toxicities: a collaborative position paper. Annals of oncology : official journal of the European Society for Medical Oncology.

[14] Michot JM, Bigenwald C, Champiat S, Collins M, Carbonnel F, Postel-Vinay S, et al. Immune-related adverse events with immune checkpoint blockade: a comprehensive review. European journal of cancer (Oxford, England : 1990). 2016;54:139–48. 10.1016/j.ejca.2015.11.016 26765102

[15] Manohar S, Kompotiatis P, Thongprayoon C, Cheungpasitporn W, Herrmann J, Herrmann SM (2019). Programmed cell death protein 1 inhibitor treatment is associated with acute kidney injury and hypocalcemia: meta-analysis. Nephrology, dialysis, transplantation : official publication of the European Dialysis and Transplant Association - European Renal Association.

[16] Motzer RJ, Tannir NM, McDermott DF, Aren Frontera O, Melichar B, Choueiri TK (2018). Nivolumab plus Ipilimumab versus Sunitinib in advanced renal-cell carcinoma. N Engl J Med.

[17] Cortazar FB, Marrone KA, Troxell ML, Ralto KM, Hoenig MP, Brahmer JR (2016). Clinicopathological features of acute kidney injury associated with immune checkpoint inhibitors. Kidney Int.

[18] Fadel F, El Karoui K, Knebelmann B (2009). Anti-CTLA4 antibody-induced lupus nephritis. N Engl J Med.

[19] Daanen RA, Maas RJH, Koornstra RHT, Steenbergen EJ, van Herpen CML, Willemsen A. Nivolumab-associated Nephrotic Syndrome in a Patient With Renal Cell Carcinoma: A Case Report. Journal of immunotherapy (Hagerstown, Md : 1997). 2017;40(9):345–8. 10.1097/CJI.0000000000000189 28961608

[20] Kidd JM, Gizaw AB (2016). Ipilimumab-associated minimal-change disease. Kidney Int.

[21] Ashour T, Nakhoul G, Patil P, Funchain P, Herlitz L (2019). Immune check point inhibitor-associated glomerulonephritis. Kidney international reports.

[22] Takahashi N, Tsuji K, Tamiya H, Shinohara T, Kuroda N, Takeuchi E. Goodpasture's disease in a patient with advanced lung cancer treated with nivolumab: An autopsy case report. Lung cancer (Amsterdam, Netherlands). 2018;122:22–4. 10.1016/j.lungcan.2018.05.015 30032835

[23] Mamlouk O, Selamet U, Machado S, Abdelrahim M, Glass WF, Tchakarov A (2019). Nephrotoxicity of immune checkpoint inhibitors beyond tubulointerstitial nephritis: single-center experience. Journal for immunotherapy of cancer.

[24] Shirali AC, Perazella MA, Gettinger S (2016). Association of Acute Interstitial Nephritis with Programmed Cell Death 1 inhibitor therapy in lung Cancer patients. American journal of kidney diseases : the official journal of the National Kidney Foundation.

[25] Weiss SA, Wolchok JD, Sznol M. Immunotherapy of melanoma: facts and hopes. Clinical cancer research : an official journal of the American Association for Cancer Research. 2019. 10.1158/1078-0432.CCR-18-1550 PMC6726509 30923036

[26] Larkin J, Chiarion-Sileni V, Gonzalez R, Grob JJ, Rutkowski P, Lao CD (2019). Five-year survival with combined Nivolumab and Ipilimumab in advanced melanoma. N Engl J Med.

